# Web-based questionnaires to capture acute infections in long-term cohorts: Findings of a feasibility study

**DOI:** 10.1007/s00103-014-2049-6

**Published:** 2014-10-08

**Authors:** S. Mall, M.K. Akmatov, A. Schultze, W. Ahrens, N. Obi, F. Pessler, G. Krause

**Affiliations:** 1 Department for Epidemiology, Helmholtz Centre for Infection Research, Inhoffenstraße 7, 38124 Braunschweig, Germany; 2 Molecular Epidemiology Research Group, Max-Delbrück-Center for Molecular Medicine, Berlin, Germany; 3 Leibniz Institute for Prevention Research and Epidemiology—BIPS, Bremen, Germany; 4 University Cancer Center Hamburg, University Medical Center Hamburg-Eppendorf, Hamburg, Germany; 5 TWINCORE Centre for Experimental and Clinical Research, Hannover, Germany; 6 Hanover Medical School, Hannover, Germany

**Keywords:** Web-based data collection, Prospective study, Respiratory infections, Gastrointestinal infections, Influenza-like illness, Web-basierte Datenerfassung, Prospektive Studie, Machbarkeitsstudie, Infektionen, Respiratorische Infektionen, Gastrointestinale Infektionen, Influenza-ähnliche Erkrankung

## Abstract

**Background:**

Incidence of acute respiratory infections (ARI) and gastrointestinal infections (GII) are difficult to assess due to high frequency episodes, limited severity and short duration. Retrospective assessments therefore are particularly prone to *recall bias*, while prospective assessment with conventional questionnaires requires high discipline from participants which is difficult to maintain over longer time periods. Web-based questionnaires (WQ) allow integration of a recall system and thus carry the potential to prospectively capture acute infections. We investigated the feasibility of a weekly WQ assessing symptoms of ARI and GII among participants of the German National Cohort (GNC).

**Material and methods:**

In the study centres Hamburg and Bremen of the GNC participants of the Pretest 1 phase (September to November 2011) were invited to additionally take part in this feasibility study testing the WQ. Every Monday participants received an e-mail, containing a link to the WQ, asking for occurrence of ARI or GII symptoms during the past 7 days. The study took place from the beginning of February until mid-July 2012. We calculated the overall proportion of participation, weekly participation and the number of weekly reports per participant and we estimated incidences of ARI, ILI and GII.

**Results:**

Of 200 Pretest 1 participants 171 (86 %) reported having an email address and thus were eligible for the web-based study. A total of 167 (98 %) agreed to participate. Participants of the web-based study were younger and better educated than non-participants. Access to Internet decreased with increasing age. Of the 167 participants in the feasibility study, 144 (86 %) responded at least once during the study period of 23 weeks, 5 persons (3 %) had non-functioning email addresses and 18 (11 %) did not respond at all. The weekly response varied between 62 % and 81 %, the median was 74 % (IQR: 71–77 %). Weekly median reports per person were 20 (IQR: 14–22; range 1–23). More than 90 % of participants responded during the first 3 days. The following mean incidence rates were found: ARI, 12 %; ILI, 0.49 %; and GII, 3 %.

**Conclusion:**

Use of WQ in prospective studies seems well possible, as Internet access is frequent among study participants and major technical problems did not occur. We observed high participation during the study period of 6 months and low drop out numbers. Participants of the web-based study were slightly younger and better educated than non-participants, so *selection bias* is possible and must be kept in mind when discussing generalizability of the results.

## Abstract (de)

**Hintergrund:**

Die Inzidenz akuter respiratorischer Atemwegs- (ARE) und gastrointestinaler (GIE) Erkrankungen ist schwierig zu erfassen, da Krankheitsepisoden häufig auftreten und diese überwiegend einen kurzen und leichten Verlauf haben. Retrospektive Befragungen sind daher oft anfällig für *recall bias*, während bei einem prospektiven Vorgehen von den Teilnehmern eine große Disziplin über einen längeren Zeitraum gefordert ist. Web-basierte Fragebögen (WF) erlauben die Integration eines Erinnerungssystems mit kurzen Intervallen und sind potentiell gut geeignet, um häufig auftretende, akute Infektionen über einen längeren Zeitraum zu erfassen. Wir untersuchten, ob Probanden im Rahmen der Nationalen Kohorte (NAKO) bereit und in der Lage sind, ARE- und GIE-Symptome zu erfassen, indem sie über wöchentlich versandte Erinnerungs-E-Mails einen Link zu einem Online-Fragebogen zugeschickt bekommen.

**Material und Methoden:**

In den Studienzentren Hamburg und Bremen wurden Teilnehmer des 1. Pretests (09–11/2011) eingeladen, zusätzlich an der Machbarkeitsstudie zum WF teilzunehmen. Jeden Montag erhielten die Teilnehmer eine E-Mail mit einem Link zum WF, in dem sie nach ARE- oder GIE -Symptomen in den vorangegangenen 7 Tagen gefragt wurden. Die Studie wurde von Februar bis Mitte Juli 2012 durchgeführt. Wir untersuchten den Anteil der Teilnehmer insgesamt, die wöchentliche Teilnahme, die wöchentlichen Rückmeldungen/Teilnehmer und berechneten Inzidenzen für ARE, Influenza-ähnliche Erkrankung (ILI) und GIE.

**Ergebnisse:**

Von 200 Teilnehmern des Pretests erfüllten 171 (86 %) die Voraussetzungen (Zugang zu internetfähigem PC mit funktionierender Email Adresse) für die Teilnahme an der Machbarkeitsstudie. Von diesen 171 Teilnehmern stimmten 167 (98 %) der Teilnahme zu. Teilnehmer waren etwas jünger als Nichtteilnehmer und besser gebildet. Der Zugang zum Internet nahm mit zunehmendem Alter ab. Fünf Teilnehmer (3 %) hatten keine funktionierende E-Mail-Adresse, 18 (11 %) antworteten nicht, 144 (86 %) antworteten mindestens einmal während der gesamten Studie (23 Wochen). Die wöchentlichen Rückmeldungsraten variierten zwischen 62 % und 81 %, der Median betrug 74 % (Interquartil-Bereich 71–77 %). Der Median für die Anzahl der Rückmeldungen/Teilnehmer lag bei 20 (Interquartil-Bereich 14–22; Spannweite 1–23). Über 90 % der Teilnehmer meldeten sich innerhalb der ersten 3 Tage zurück. Die mittlere Inzidenz für ARE und ILI betrug 12 % und 0,49 %, für GIE 3,0 %.

**Schlussfolgerung:**

Der Einsatz von WF in der NAKO erscheint gut durchführbar, die Anzahl der Studienteilnehmer mit Internetanschluss war hoch, es traten keine größeren technischen Probleme in der Durchführung auf. Die Teilnahme blieb während der 6-monatigen Studie hoch, die Anzahl von Ausfällen gering. Die Teilnehmer an der Machbarkeitsstudie waren jünger und besser gebildet als Nichtteilnehmer, daher muss ein möglicher *selection bias* berücksichtigt werden, wenn es um die Übertragbarkeit der Ergebnisse auf die Allgemeinbevölkerung geht.

Acute respiratory (ARI) and gastrointestinal infections (GII) have an important share of the overall burden of disease [1]. However, the acute onset, relative harmlessness and short duration of the majority of symptomatic episodes, nearly always leading to *restitutio ad integrum*, make it difficult for most people to remember symptoms, number and intensity of episodes during a certain time period. Assessments of the magnitude and intensity of ARI and GII are thus often flawed by recall bias^1^ and difficult to examine in long-term cohort studies. Previous research on acute infections used different methods to capture symptomatic episodes, such as daily diaries [2], phone calls [3], emails [4], or short text message service (SMS) [5–7]. None of these methods turned out to be simple and efficient with some resulting in poor compliance, while others were costly and time consuming. A solution could be to use a web-based tool to prospectively capture episodes of acute infections. The advantages of such tools have been described by several authors [8, 9]. They include fast data transfer, improved data quality (validation checks and subsequent alerts in case of false/incomplete answers can be built in), also nonresponse and “don’t know” answers are reported to be less prevalent in web-based questionnaires compared with postal questionnaires [10]. Web-based questionnaires are returned more rapidly than postal questionnaires, most respondents completing the questionnaire within a few days [11]. Automated e-mail reminders and invitations for follow-up questionnaires can be sent to study participants, although follow-up of “bounce-back” (undeliverable) e-mails may be time-consuming [12]. Because of their advantages, web-based tools have mainly been used in surveys but are now also increasingly used in epidemiological studies [13, 14]. Few investigators have applied web-based tools, mostly involving patients with chronic medical conditions [15]. Many studies were hospital-based or were conducted in specific population groups such as health care workers, birth cohorts or sentinel general practitioner sites. For acute infections prospective web-based data collection has rarely been used in truly population-based studies.

In the GNC, it is planned to follow-up participants over a long time period, so choosing an instrument that may be little time consuming and not too demanding may be particularly valid in terms of participation. Taking all aspects into account, we decided that the tool to prospectively gather information on ARI and GII should be able to collect data rapidly while optimizing data quality and minimizing recall bias. It should also be user friendly and not use much of the participants’ time, as participation in studies decreases with increasing time and effort required for completing the questionnaires [16].

Knowing that the GNC aims to maintain continual participation over a long period of time, we felt that a web-based questionnaire would best fulfil these requirements and have a less discouraging effect on potential participants.

Thus, before using the web-based questionnaire as a new tool in larger and more sophisticated studies on infectious diseases including the GNC (e.g. self-collected sampling combined with questionnaires), we conducted a feasibility study to assess technical realisation and potential participation over time.

## Methods

### Study population, recruitment and eligibility

The web-based feasibility study on acute infections was conducted in two (Bremen and Hamburg) of the 17 study centers participating in Pretest 1. Potential study participants for Pretest 1 were randomly selected from population registries and invited by postal mail to participate and were offered an incentive of 25 € (Hamburg) or 30 € (Bremen) [17]. No further money or incentives were offered for the additional participation in the web-based study. During the Pretest 1 phase, reminders were sent to those participants from whom no response was received. Individuals whose phone number could be ascertained from the public telephone directory were reminded by phone call (38 % in Bremen and 43 % in Hamburg). In addition, participants in Bremen received personal results of the medical examinations. Each study center recruited 100 participants for the Pretest 1 phase. This study was conducted during September–November 2011 in both cities. The participants of the Pretest 1 who reported to have an email address and access to the Internet were eligible to participate in the web-based study. They were asked whether they would like to also participate in a prospective web-based study on acute infections. Those who consented to the web-based study provided their email address. The web-based study lasted 23 weeks between February and July 2012. Every Monday morning, the participants received an email containing brief information about the study and a link to the web questionnaire. Each week participants were asked to report a *new* episode of acute respiratory and gastrointestinal infections occurring in the last week and asked specifically for symptoms.

### Questionnaire on acute infections and case definitions

We developed a short questionnaire containing two questions on symptoms of ARI and GII using open source software (www.limesurvey.org). The first question addressed 12 symptoms (sneezing, runny nose, plugged nose, dry cough, cough with expectoration, sore throat, headache, muscle and/or joint pain, malaise, fever > 38.5 °C and/or chills, dyspnea with rhonchus and strong bilateral calf pain induced on stretching including difficulty to walk because of this calf pain) and included one free text field for additional symptoms. The second question addressed two symptoms of gastrointestinal infections (diarrhea and vomiting).

To be able to compare our data with other available data on respiratory infections, we used the Robert Koch Institute criteria for ARI and ILI [18]. An ARI was defined as subjectively reported respiratory illness with new onset of fever or cough (dry or with expectoration) or sore throat. An ILI was defined as subjectively reported respiratory illness and fever plus presence of cough or sore throat. A recurrent episode of ARI/ILI was defined when participants had at least one week without symptoms since the last reported episode of ARI/ILI. We removed first reports of all participants since participants tended to participate in the web-based study when they developed an episode of ARE or ILI.

An acute gastroenteritis was defined as having had loose stools ≥ 3 times or at least one episode of vomiting in 24 h [19]. A recurrent episode of gastroenteritis was defined when participants had a 2-week symptom-free period and reported a new episode of loose stools or vomiting [20].

### Statistical analysis

First, Pretest 1 participants were divided into two groups, eligible and non-eligible for participation in the web-based study and then compared in terms of basic sociodemographic data. Eligibility was defined as having an email address and having access to a personal computer with Internet connection. Then we calculated the proportion of pretest participants who agreed to participate in the prospective web-based study (participation proportion). Furthermore, we calculated the proportion of individuals who filled in the web-based questionnaire each week (weekly participation) stratified by study center. The number of weekly reports per participant was calculated (individual participation over time). Finally, we calculated weekly infection rates by dividing the total number of individuals who reported a new infection episode by the total number of individuals who responded in a particular week. We applied a 3-week moving average method and presented incidences of acute infections graphically. The differences between categorical variables were examined by the chi-squared testor Fisher's exact test.

### Ethical and data safety considerations

Ethical approval was obtained from the Ethics Committees of the State Board of Physicians in Bremen and in Hamburg. Written informed consent for the whole study and specific consent for the prospective web-based study was obtained from all participants. Participants’ email addresses were stored at a separate server of the Helmholtz Centre for Infection Research (HZI) for the duration of the study and deleted after completion of the study.

## Results

### Participants eligible and not eligible for the web-based study

**Fig. 1:**
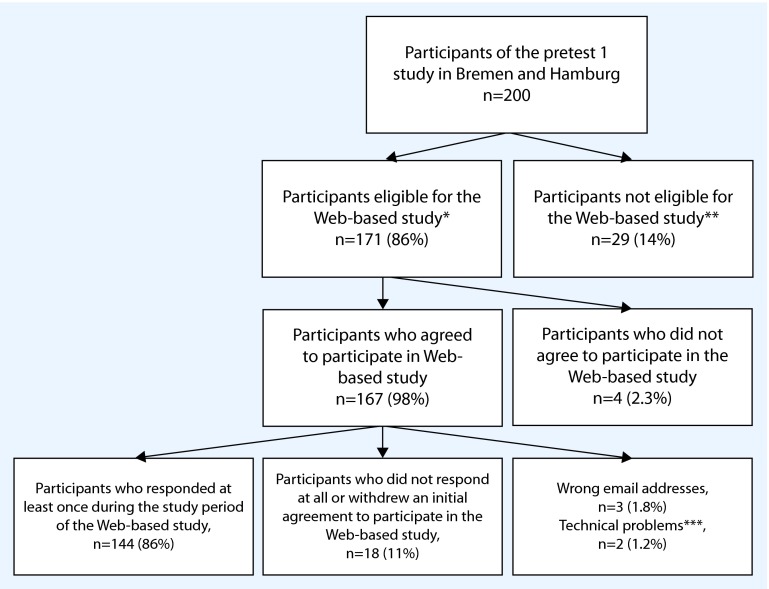
Flow chart of the study population (* Individuals with email address, ** Individuals without email address, *** Emails could not be delivered due to technical problems)

Of 200 participants of the Pretest 1 study in Bremen and Hamburg, 171 (86 %) reported having an email address and 29 (14 %) did not have an email address (Fig. 1). The availability of email addresses decreased with increasing age; for example, all participants in the age groups “20–29” and “30–39” reported having an email address, while only about 60 % in the age groups “60–69” did so. Compared to participants eligible for the web-based study, non-eligible participants were significantly older and had lower education (Table 1, columns 3 and 4). In addition, the proportion of individuals with diabetes was higher among non-eligible participants than among eligible participants (*p* = 0.36).

**Table 1:** Characteristics of the individuals participated in the web-based study compared to participants of the Pretest 1 study of the German National Cohort

Characteristics	Participants of Pretest 1 study *n (%)*	Participants eligible for the web-based study^a^ *n (%)*	Participants not eligible for the web-based study^b^ *n (%)*	*p* value*	Participants of the web-based study *n (%)*
**Sex**				0.8	
Male	87 (44)	75 (44)	12 (41)		63 (44)
Female	113 (56)	96 (56)	17 (59)		81 (56)
**Age groups**				< 0.0001	
< 20	1 (0.5)	1 (0.6)	0 (0)		1 (0.69)
20–29	32 (16)	32 (19)	0 (0)		27 (19)
30–39	27 (13)	27 (16)	0 (0)		24 (17)
40–49	39 (19)	35 (21)	4 (14)		29 (20)
50–59	43 (22)	40 (23)	3 (10)		35 (24)
60–69	58 (29)	36 (21)	22 (76)		28 (19)
**Education level**				0.003	
Low	24 (13)	16 (10)	8 (32)		12 (9.2)
Middle	53 (29)	44 (28)	9 (36)		36 (28)
Higher	105 (58)	97 (62)	8 (32)		83 (63)
**Country of birth**				0.91	
Germany	171 (86)	146 (85)	25 (86)		125 (87)
Other	29 (14)	25 (15)	4 (14)		19 (13)
**Diabetes**				0.36	
Yes	13 (6.5)	10 (5.8)	3 (10)		6 (4.2)
No	187 (93.5)	161 (94)	26 (89)		138 (95.8)

^a^Individuals with email address

^b^Individuals without email address*Fisher’s exact test

### Participation in the prospective web-based study

**Fig. 2:**
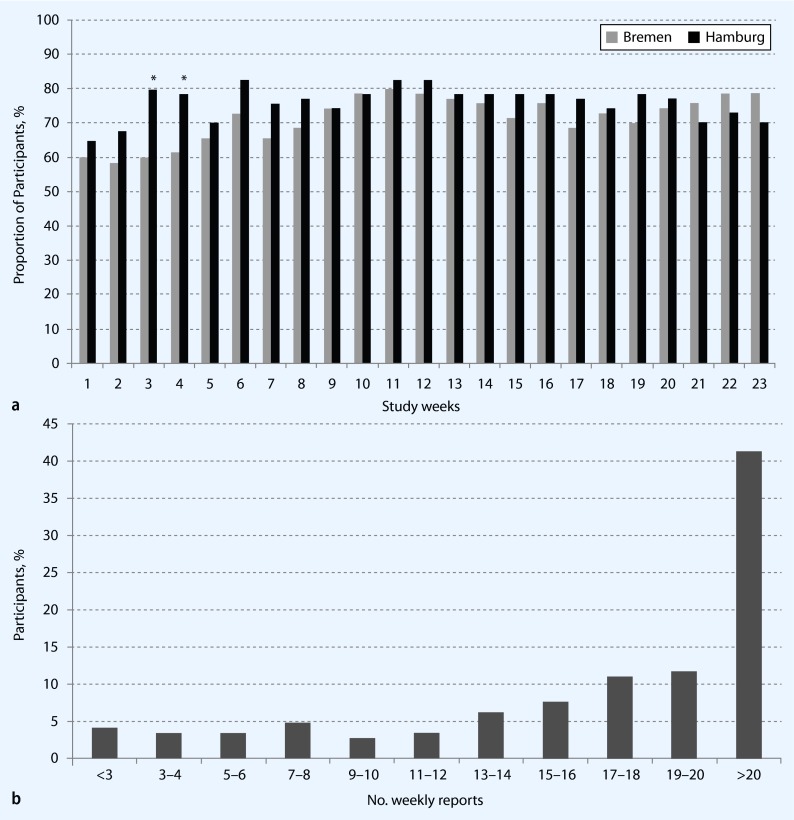
Participation in the web-based study on acute infections. **a** Proportion of individuals who participated each week in the web-based study (* *p* < 0.05). **b** Number of weekly reports per participant (*n* = 144)

Of 171 individuals eligible to participate in the web-based study, 167 (98 %) agreed to participate. Thus, 84 % (167/200) of the Pretest 1 participants at these locations were both eligible and agreed to participate in the web-based study. Of these 167 participants, 144 (86 %) responded at least once during the study period of 23 weeks (Fig. 1). When considering the total sample of Pretest 1 participants, 72 % (144/200) responded at least once during the study period. Three percent of email addresses (5/167) did not function (wrong email addresses or emails could not be delivered) and 11 % (18/167) did not respond at all. Over the study period the proportion of participants who responded each week varied between 62 % and 81 % (median: 74 %; IQR: 71–77 %), the respective differences between Hamburg and Bremen were not significant, except in study weeks 3 and 4 (Fig. 2a). More than 90 % of participants responded weekly, within the first 3 days. The median number of weekly reports per participant was 20 (IQR 14–22; range 1–23; Fig. 2b).

### Acute infections

**Fig. 3:**
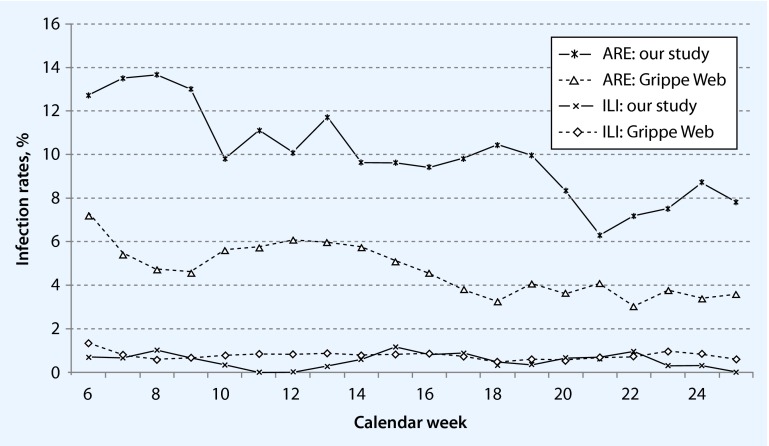
ARE/ILI infection rates by week: feasibility study versus GrippeWeb, %

Mean incidence over the whole study time were: ARI, 12 % (range 3.8–17.5 %); ILI, 0.49 % (range 0–1.9 %); and acute gastroenteritis, 3 % (range 0.93–8.9 %). The weekly incidence rates of ARI were about two times higher in our data than incidence rates in the GrippeWeb—a syndromic surveillance of acute respiratory infections in the general population of Germany operated by the Robert Koch Institute (Fig. 3). The incidence curve had a similar shape over the study period as in GrippeWeb. The weekly incidence curves of ILI were similar to the data from the syndromic surveillance system during the same period of time.

## Discussion

Germany has good prerequisites for web-based studies with its high coverage of Internet access in the general population. In 2012, approximately 80 % of the German households had a personal computer with Internet access [21]. We examined the feasibility of using a web-based tool to capture acute infections in a longitudinal study embedded in a larger population-based study. We observed that the majority of participants (171/200; 86 %) reported having an email address and thus were eligible to participate in the web-based study. Almost all of the participants in the Pretest 1 study (167/171; 98 %) agreed to also take part in the feasibility study as well—without additional incentives offered. There were some differences between individuals eligible and not eligible for the web-based study in terms of selected sociodemographic characteristics, i.e. eligible individuals were younger and had a higher education level (62 % vs. 36 %). We found similar prevalences of type 2 diabetes—a chronic disease which we had chosen for representativeness—in the general population: about 7.2 % [22] and 6.5 % among the Pretest 1 participants.

How could age composition in the two groups affect our study? We may have selected younger, better educated and healthier people as our future participants, so this bias needs to be kept in mind when it comes to generalizability of the results. It is also unclear to what extent Internet competency of participants may decrease as they get older which may become important as we intend to follow participants up for several decades within the GNC. On the other hand, Internet competency could improve in the same age groups over the coming years. As our study was only conducted over just 6 months, we can only speculate about these issues.

We observed a high weekly participation between 62 and 81 %, which remained constant over 6 months, suggesting that individuals remain highly motivated once they agreed to participate in the web-based study. We found this promising, although we cannot conclude from these results a continuously high participation in the future, especially not when thinking of follow-up periods of several years. A weekly reporting interval seems to be possible and not too burdensome for the participants. To assess incidence of acute infections which are of short duration, weekly or bi-weekly intervals seem to be reasonable.

There were only minor differences in weekly participation between the two study centers. We saw that 11 % of individuals who initially agreed to participate in the web-based study did not respond at all and technical problems occurred in 3 % of cases (e.g. not functioning email address). The proportion of about 11 % dropouts without technical problems may be explained by the time gap of several months between the initial recruitment of participants of the Pretest 1 study (September 2011) and the start of our feasibility study (February 2012).

To see whether reported incidences of ILI and ARI were valid, we compared our weekly incidences of ILI and ARI with the data from the GrippeWeb of RKI using the same case definitions. We found similar infection rates for ILI during the same time (February–July 2012), place (Hamburg/Bremen) and age range (20–69 years) (Fig. 3). For ARI, we observed a similar trend as GrippeWeb, but incidence rates were higher in our sample.

For gastrointestinal infections, there is no national syndromic surveillance system available for comparison to test whether our results were plausible. In a recent cross-sectional survey in Germany [23], a 4-week incidence of acute gastrointestinal illness of 7.3 % was observed, corresponding to a weekly incidence of about 1.8 %. We observed weekly incidence rates ranging between 0.9 % and 8.9 % in our sample.

If the web-based tool will be incorporated as a data collection tool for infectious diseases in the GNC, we should be able to observe whether this high participation of over 60 % can be kept up continuously or only repeatedly for short periods of time. We may find that this tool is mostly useful for research questions covering a prospective observation period of only a few years as opposed to several decades

We also observed an important point: although the importance of data safety and privacy are widely discussed in the media, participants were not reluctant to share their data via the web. Finally, the remuneration offered for participation in the GNC seemed to be sufficient to also cover participation in the web-based study. There were no additional incentives offered and yet participation in the feasibility study remained constantly high throughout the study period.

## Conclusion

Web-based questionnaires could offer a promising chance for prospective data collection of infectious diseases within the setting of the GNC. They may be especially valuable for repeatedly occurring infections of short duration which are prone to recall bias. There is, however, still a danger of selection bias, as certain groups (e.g. elderly, less educated) may be underrepresented simply because of lower access to the Internet. The validity of data gathered through this new tool within the GNC needs to be evaluated in the future; however first results from this feasibility study seem to be comparable with the data from other sources when looking at incidence data for ILI or GII.

We believe web-based questionnaires can be considered a valuable data collection tool within the GNC that can be integrated easily and complement other forms of data collection as long as its limitations—specifically selection bias—are kept in mind.

## Footnotes

1 Recall bias is a systematic error due to differences in accuracy or completeness of recall to memory of past events or experiences.
